# MRI-targeted biopsy cores from prostate index lesions: assessment and prediction of the number needed

**DOI:** 10.1038/s41391-022-00599-2

**Published:** 2022-10-08

**Authors:** Nick Lasse Beetz, Franziska Dräger, Charlie Alexander Hamm, Seyd Shnayien, Madhuri Monique Rudolph, Konrad Froböse, Sefer Elezkurtaj, Matthias Haas, Patrick Asbach, Bernd Hamm, Samy Mahjoub, Frank Konietschke, Maximilian Wechsung, Felix Balzer, Hannes Cash, Sebastian Hofbauer, Tobias Penzkofer

**Affiliations:** 1 Department of Radiology, Charité University Hospital Berlin, corporate member of Freie Universität Berlin and Humboldt-Universität zu Berlin, Berlin, Germany; 2 Berlin Institute of Health at Charité-Universitätsmedizin Berlin, BIH Biomedical Innovation Academy, Berlin, Germany; 3 Department of Pathology, Charité University Hospital Berlin, corporate member of Freie Universität Berlin and Humboldt-Universität zu Berlin, Berlin, Germany; 4 Department of Urology, Medizinische Hochschule Hannover, Hannover, Germany; 5 Institute of Biometry and Clinical Epidemiology, Charité University Hospital Berlin, corporate member of Freie Universität Berlin and Humboldt-Universität zu Berlin, Berlin, Germany; 6 Institute of Medical Informatics, Charité University Hospital Berlin, corporate member of Freie Universität Berlin and Humboldt-Universität zu Berlin, Berlin, Germany; 7 Department of Urology, University Hospital Magdeburg, Magdeburg, Sachsen-Anhalt Germany; 8 Department of Urology, Charité University Hospital Berlin, corporate member of Freie Universität Berlin and Humboldt-Universität zu Berlin, Berlin, Germany

**Keywords:** Prostatic diseases, Cancer screening

## Abstract

**Background:**

Magnetic resonance imaging (MRI) is used to detect the prostate index lesion before targeted biopsy. However, the number of biopsy cores that should be obtained from the index lesion is unclear. The aim of this study is to analyze how many MRI-targeted biopsy cores are needed to establish the most relevant histopathologic diagnosis of the index lesion and to build a prediction model.

**Methods:**

We retrospectively included 451 patients who underwent 10-core systematic prostate biopsy and MRI-targeted biopsy with sampling of at least three cores from the index lesion. A total of 1587 biopsy cores were analyzed. The core sampling sequence was recorded, and the first biopsy core detecting the most relevant histopathologic diagnosis was identified. In a subgroup of 261 patients in whom exactly three MRI-targeted biopsy cores were obtained from the index lesion, we generated a prediction model. A nonparametric Bayes classifier was trained using the PI-RADS score, prostate-specific antigen (PSA) density, lesion size, zone, and location as covariates.

**Results:**

The most relevant histopathologic diagnosis of the index lesion was detected by the first biopsy core in 331 cases (73%), by the second in 66 cases (15%), and by the third in 39 cases (9%), by the fourth in 13 cases (3%), and by the fifth in two cases (<1%). The Bayes classifier correctly predicted which biopsy core yielded the most relevant histopathologic diagnosis in 79% of the subjects. PI-RADS score, PSA density, lesion size, zone, and location did not independently influence the prediction model.

**Conclusion:**

The most relevant histopathologic diagnosis of the index lesion was made on the basis of three MRI-targeted biopsy cores in 97% of patients. Our classifier can help in predicting the first MRI-targeted biopsy core revealing the most relevant histopathologic diagnosis; however, at least three MRI-targeted biopsy cores should be obtained regardless of the preinterventionally assessed covariates.

## Introduction

In Europe and North America, prostate cancer (PCa) is the most common cancer in men [1, 2]. Despite advances in PCa imaging with magnetic resonance imaging (MRI) and prostate-specific membrane antigen (PSMA) positron emission tomography (PET), histopathologic verification remains the gold standard for establishing the diagnosis of PCa [3, 4]. Thus, in Europe and North America alone, over 2 million prostate biopsies are performed every year [5].

Obtaining a prostate biopsy is considered if prostate-specific antigen (PSA) is elevated (≥4 ng/ml), an abnormal increase in PSA levels compared to baseline is observed in serial examinations, and if there are pathologic findings in the digital rectal examination [6]. However, to reduce overdiagnosis and overtreatment, international guidelines advocate multiparametric MRI (mpMRI) in biopsy-naïve men with suspected PCa before prostate biopsy [7–9]. Even though severe complications are rare, infection, hematospermia, hematuria, and rectal bleeding may occur [10, 11]. Combined MRI-targeted and systematic prostate biopsy detects up to 70% of clinically significant (cs)PCa with a Gleason grade group ≥2 [12]. The 2019 European Association of Urology (EAU) guidelines, which are endorsed by the PI-RADS steering committee, suggest the use of combined systematic and targeted biopsies in biopsy-naive men with intermediate- or high-likelihood MRI findings and recommend omitting systematic sampling in those with prior negative biopsy results [13]. In patients with low-likelihood MRI findings, re-biopsy should only be performed based on shared decision making with the patient [14]. Nonetheless, the National Institute for Health and Care Excellence (NICE) guidelines suggest considering whole-prostate mapping biopsies in high-risk patients with negative findings at prior biopsy even if MRI findings indicate low likelihood of csPCa [8].

In a systematic prostate biopsy, 10 to 12 transrectal ultrasound (TRUS)-guided tissue samples are obtained: one each on both sides of the prostate in the base, midgland and apex, and four to six lateral samples covering the peripheral zone [15–17]. It has been shown that obtaining more than 12 systematic tissue cores does not result in a significantly better cancer detection rate, but more adverse events [18, 19]. Suspicious areas on mpMRI can be targeted with MRI/TRUS fusion biopsy, percutaneous biopsy during MRI, or TRUS biopsy with visual review of MRI (cognitive biopsy) [20]. Each target should be sampled at least twice, and more tissue cores should be obtained depending on size, location, and confidence of targeting [21].

In many cases, imaging studies detects more than one suspicious lesion [22]. Therefore, an index lesion is usually defined as an abnormal focal area that is detected on mpMRI and that most likely harbors PCa [23]. Detecting the index lesion on initial mpMRI is important as it determines disease progression in multifocal disease while secondary lesions seem not contribute to clinical outcome [24]. Consequently, some studies suggest that, when focal therapy is performed, targeting the index lesion alone may be sufficient [25].

Saturation biopsy is defined by a sampling technique, whereby inclusion of additional biopsy cores would not increase the detection rate of PCa [26]. PI-RADS has proven to have good sensitivity when a score of ≥3 is considered as a positive test [27]. Especially for PI-RADS > 3 lesions, detection of csPCa can be improved by increasing the number of MRI-targeted biopsy cores [28]. A study of van der Leest found that, when MRI-guided biopsy of prostate lesions is performed, “local saturation” by an additional four perilesional cores can improve detection of csPCa [29]. However, a meta-analysis of Mazzone et al. demonstrated that the positive predictive value of highly suspicious lesions detected on mpMRI is not high enough to omit systematic prostate sampling [30]. Combined systematic and targeted biopsies show the highest concordance with the final histopathologic diagnosis after radical prostatectomy, and the total number of cores seems to be an independent predictor of concordance [31].

Several studies show that, in MRI-targeted biopsy, obtaining additional cores (“focal saturation”) increases biopsy yields [32, 33]. Still, there is an ongoing controversy about the optimal number of MRI-targeted biopsy cores to be obtained from the index lesion. On the one hand, some studies demonstrate that, compared with only two targeted cores, five targeted cores can increase the detection of csPCa. For example, Lu et al. have shown that obtaining only two MRI-targeted cores achieves 25% lower detection of csPCa compared to obtaining five MRI-targeted cores [34]. In addition, a study of Calio et al. has shown that more targeted biopsy cores reduce the probability of needing to upgrade the risk category after prostatectomy [35]. On the other hand, it has been reported that the first two biopsy cores already detect the highest Gleason score and comprise the first clinically significant cancer core in 88% of cases [36]. Moreover, a higher Gleason score is assigned in only 10% of cases when a second targeted biopsy core is taken compared to only one, while an increasing number of biopsy cores is associated with a higher risk of diagnosing clinically nonsignificant (cns)PCa [37, 38].

Predicting the first MRI-targeted biopsy core that will reveal the most relevant histopathologic diagnosis could be of high clinical relevance. A possible prediction model that can be used in this setting is the Bayes classifier. This classifier is a probabilistic machine learning model that is used to discriminate different outcomes (e.g., needed number of MRI-targeted biopsy cores) and is based on certain features (e.g., PI-RADS score and lesion size) [39].

We therefore conducted a study in which we assessed possible influencing factors for the first biopsy core revealing the most relevant histopathologic diagnosis in a large population of patients with suspected PCa who underwent MRI-targeted biopsy with sampling of at least three tissue cores from the index lesion and in whom the chronological order in which the MRI-targeted biopsy cores were obtained was available. The hypothesis of this study is that the number of MRI-targeted biopsy cores needed to establish the most relevant histopathologic diagnosis of the prostate index lesion detected on mpMRI depends on different variables including the PI-RADS score, PSA density, lesion size, zone, and location. Our aim thus is to systematically analyze how many MRI-targeted cores of the index lesion are needed to establish the most relevant histopathologic diagnosis and to build a prediction model to forecast the number of MRI-targeted biopsy cores. As the optimal number of MRI-targeted biopsy cores is uncertain in clinical routine, our results could aid in predicting and justifying the number to be obtained.

## Material and methods

### Study design

The study was approved by the institutional review board (Ethikkommission der Charité—Universitätsmedizin Berlin, approval number EA1/271/16), which waived the need for patient consent. This single-center, retrospective cohort study was designed to identify the number of MRI-targeted prostate biopsy cores needed to establish the most relevant histopathologic diagnosis of the index lesion. The study was in compliance with the Declaration of Helsinki.

### Patient population, patient characteristics, and data collection

**Fig. 1:**
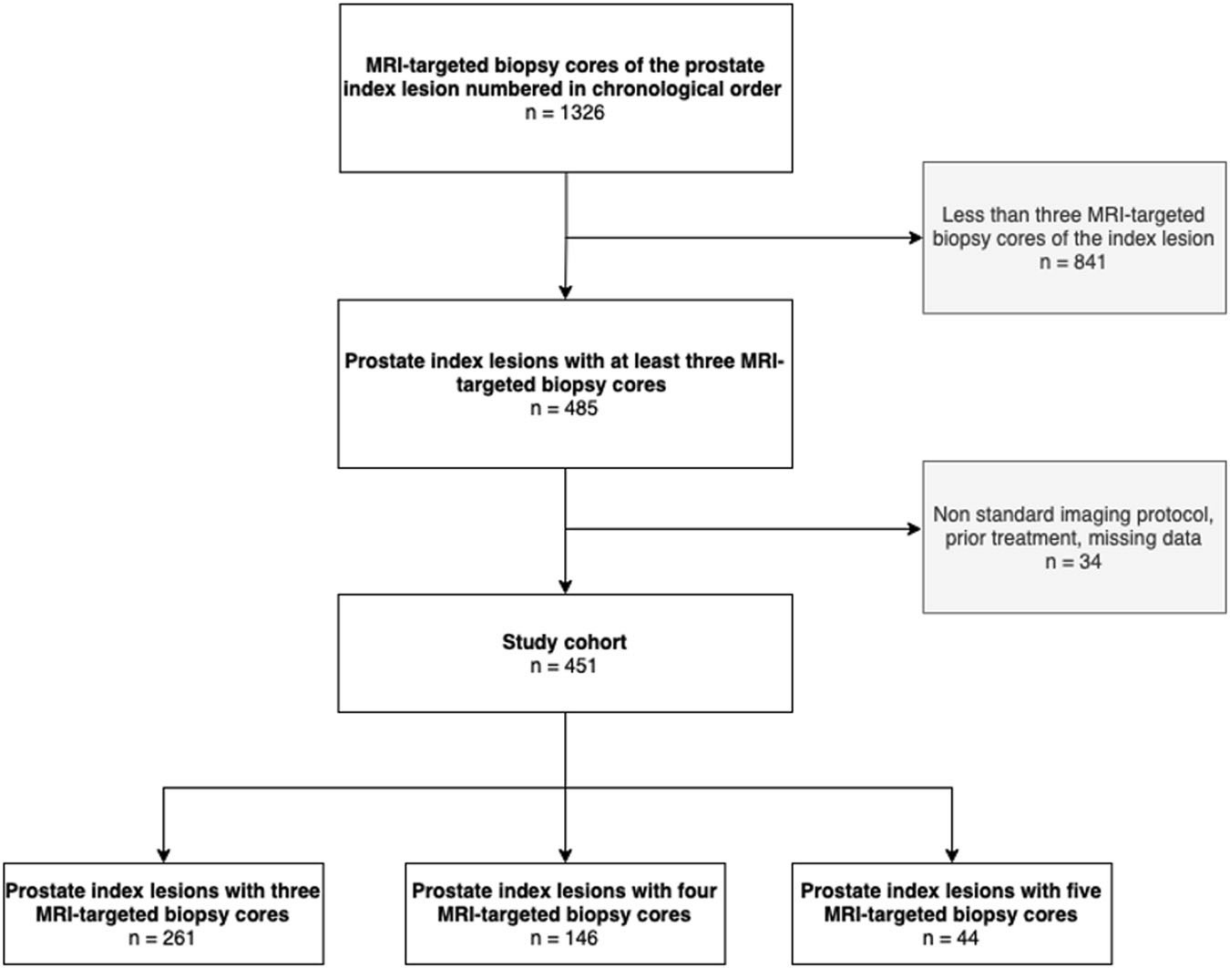
Flowchart of patient inclusion and exclusion and final study population (patients with 3 to 5 MRI-targeted core biopsies). PCa prostate cancer. MRI magnetic resonance imaging, *n* number.

The study included patients with suspected PCa who underwent mpMRI of the prostate prior to biopsy between September 2013 and April 2017 and in whom the core sampling sequence had been recorded. The index lesion was defined as an abnormal focal area detected on mpMRI and most likely harboring PCa. Lesions of all sizes were considered, but when two equivocal suspicious lesions were present, the larger lesion was defined as index lesion. The decision to obtain MRI-targeted biopsies from lesions rated PI-RADS 2 was made at the discretion of the treating urologist and in agreement with the patient. All patients underwent 10-core systematic biopsy, and at least three MRI-targeted biopsy cores using MRI/TRUS fusion were sampled from the index lesion detected on mpMRI. All biopsy cores were obtained using one of two biopsy devices (Aplio 500, Toshiba, Otawara, Japan or HI VISION Preirus, Hitachi Medical Systems, Tokyo, Japan) and 18-Gauge needles. All MRI-targeted biopsies were performed according to clinical standard of the treating urologists and aimed at the suspicious areal detected on mpMRI. Targeted tissue samples were taken from central and peripheral parts within the index lesion to ensure focal saturation. No systematic sampling of more peripheral parts outside the detected the MRI lesion (“penumbra”) was performed. During the biopsy, the MRI-targeted tissue cores were numbered 1–5 in the order of sample and stored in separate containers to ensure correct assignment for subsequent histopathologic analysis. To reduce confounders, biopsies of additional suspicious lesions (other than the index lesion) were not included in the analysis. All biopsies were taken by a team of experienced urologists and uroradiologists at our tertiary university center. Patients with prior prostate-related treatment such as brachytherapy, radiation, or hormonal treatment were excluded as were patients with missing data or use of a nonstandard imaging protocol. All data were retrieved from the patient records and clinical database. Statistical data and information on PI-RADS scores derived from mpMRI, prostate volume, PSA level, PSA density, lesion size, and zone were collected. Furthermore, the histopathologic reports on systematic and MRI-targeted biopsy cores were obtained. A flowchart of patient inclusion and exclusion is shown in Fig. 1.

### MR image acquisition

A standardized mpMRI protocol in accordance with the ESUR guidelines was used for prostate imaging in all patients [40]. The protocol included axial T1-weighted imaging (3.0 × 0.6 × 0.6 mm, 32 cm FoV), axial and coronal T2-weighted imaging (3.0 × 0.47 × 0.47 mm, 18 cm FoV), diffusion-weighted imaging (DWI) with generation of apparent diffusion coefficient (ADC) maps (3.0 × 1.4 × 1.4 mm, 17 cm FoV, with *b*-values of 0, 50, 500, 1000, and calculated *b* = 1400 s/mm^2^), and dynamic contrast-enhanced (DCE) imaging (3.0 × 1.4 × 1.4 mm, 18.6 cm FoV, 5 s temporal resolution) after intravenous injection of the Gd-based contrast medium gadobutrol (Gadovist, Bayer Healthcare) at a rate of 3 ml/s. All mpMRI examinations were performed on 3-Tesla MRI scanners equipped with multi-channel phased-array surface coils.

### Histopathologic diagnosis

The histopathologic findings of the prostate biopsies included benign / normal tissue, inflammation, and cancerous tissue, which was rated according to the Gleason score. Histopathologic analysis was performed at the Department of Pathology, Charité University Hospital. The number of the first MRI-targeted biopsy core revealing the most relevant histopathologic diagnosis of the index lesion (relevance hierarchy: highest Gleason score > prostatitis > benign prostatic hyperplasia (BPH) >normal tissue) was determined. At least three MRI-targeted biopsy cores were obtained per index lesion. Each MRI-targeted biopsy core of the index lesion was analyzed separately according to the chronologic order of sampling. Histopathologic results of MRI-targeted and systematic biopsy cores were compared using confusion matrices.

### Prediction model

Prediction analysis of the first prostate biopsy core revealing the most relevant histopathologic diagnosis for the index lesion detected on mpMRI was performed in a subgroup of patients in whom exactly three MRI-targeted biopsy cores of the index lesion were obtained. The PI-RADS score, PSA density, lesion size, zone (peripheral zone (PZ) or transition zone (TZ)), and location (apex, base or midgland) served as independent variables, and the number of the first relevant biopsy was defined as dependent variable.

### Statistical analysis

We investigated associations across variables using multinomial logistic regression analysis. Furthermore, a nonparametric regular and naïve Bayes classifier with the PI-RADS score, PSA density, lesion size, zone, and location as covariates was trained and subsequently tested [41]. The Akaike information criterion (AIC) was used to identify variables not significant for prognosis. Data were analyzed using the statistical programming package R with the add-on packages np and e1071 (R Core Team, Version 4.1.0, 2021) and SPSS (IBM, Version 27, SPSS INC., Chicago, IL, USA) at a significance level of 5% [42, 43]. All results were interpreted in an exploratory manner.

## Results

### Baseline data

A total of 451 patients with suspected prostate cancer and a mean age of 67.5 ± 7.6 years (range of 83 to 44 years) were included in this study. Thirty-four patients had to be excluded due to missing data or use of nonstandard imaging protocols. The mean PSA level was 10.2 ± 8.0 ng/ml, and mean PSA density was 0.20 ± 0.05 ng/ml/cm^3^. The index lesions were assessed by three MRI-targeted biopsy cores in 261 cases, by four MRI-targeted biopsy cores in 146 cases, and by five MRI-targeted biopsy cores in 44 cases. Overall, a total of 1587 MRI-targeted biopsy cores of index lesions were obtained. MRI-targeted index lesions ranged in size from 3.5 to 40.7 mm (15.8 ± 6.8 mm).

### PI-RADS scores and histopathologic diagnosis

All 451 prostate MRI studies were (re-)read by attending-level uroradiologists and rated according to PI-RADS v2.1. The MRI-targeted index lesion was assigned a PI-RADS score of 5 in 179 (40%) cases, a PI-RADS score of 4 in 167 (37%) cases, a PI-RADS score of 3 in 59 (13%) cases, and a PI-RADS score of 2 in 46 (10%) cases. A total of 310 MRI-targeted index lesions were in the PZ and 141 MRI-targeted index lesions were in the TZ. Relevant histopathologic findings were distributed as follows:

In the PZ, 53 (17%) of the MRI-targeted index lesions were normal prostate tissue, 27 (9%) inflammation, and 9 (3%) prostatic intraepithelial neoplasia; 54 (17%) had a Gleason score 3 + 3, 57 (18%) a Gleason score 3 + 4, 1 (0.5%) a Gleason score 3 + 5, 33 (11%) a Gleason score 4 + 3, 54 (17%) a Gleason score 4 + 4, 13 (4%) a Gleason score 4 + 5, 7 (2%) a Gleason score 5 + 4, and 2 (1%) a Gleason score 5 + 5.

In the TZ, 40 (28%) of the MRI-targeted index lesions were normal prostate tissue, 15 (10%) inflammation, and 2 (1%) prostatic intraepithelial neoplasia; 28 (20%) had a Gleason score 3 + 3, 22 (16%) a Gleason score 3 + 4, 16 (11%) a Gleason score 4 + 3, 10 (7%) a Gleason score 4 + 4, 6 (4%) a Gleason score 4 + 5, and 1 each (1%) a Gleason score 5 + 3 and 5 + 4. The histopathologic biopsy results of all MRI-targeted index lesions and the classifications according to the International Society of Urological Pathology (ISUP) are shown in Table 1.

**Table 1:** Histopathologic diagnosis of all MRI-targeted index lesions in the peripheral and transition zone of the prostate.

Peripheral zone			Transition zone		
Biopsy result	Number	Percentage (%)	Biopsy result	Number	Percentage (%)
Total	310	100	Total	141	100
Normal tissue / BPH	87	28	Normal tissue / BPH	57	40
Inflammation	2	1	Inflammation	0	0
GS 3 + 3	54	17	GS 3 + 3	28	20
GS 3 + 4	57	18	GS 3 + 4	22	16
GS 3 + 5	1	0	GS 3 + 5	0	0
GS 4 + 3	33	11	GS 4 + 3	16	11
GS 4 + 4	54	17	GS 4 + 4	10	7
GS 4 + 5	13	4	GS 4 + 5	6	4
GS 5 + 3	0	0	GS 5 + 3	1	1
GS 5 + 4	7	2	GS 5 + 4	1	1
GS 5 + 5	2	1	GS 5 + 5	0	0

ISUP Grade Group	Number	Percentage	ISUP Grade Group	Number	Percentage
Total	310	100	Total	141	100
1	143	46	1	85	60
2	57	18	2	22	16
3	33	11	3	16	11
4	55	18	4	11	8
5	22	7	5	7	5

*BPH* benign prostatic hyperplasia, *GS* Gleason score, *ISUP* International Society of Urological Pathology.

### Comparison of MRI-targeted and systematic prostate biopsies regarding PCa detection

Compared with the histopathologic findings gained from analysis of MRI-targeted biopsy cores, systematic 10-core biopsy tissue samples showed a higher Gleason score in 13 cases (4%) in the PZ and in 14 cases (10%) in the TZ. Without additional systematic biopsy, csPCa would have remained undetected in 5 cases in the PZ and in 6 cases in the TZ.

### Number of the first MRI-targeted biopsy core revealing the most relevant histopathologic diagnosis of the index lesion

**Fig. 2:**
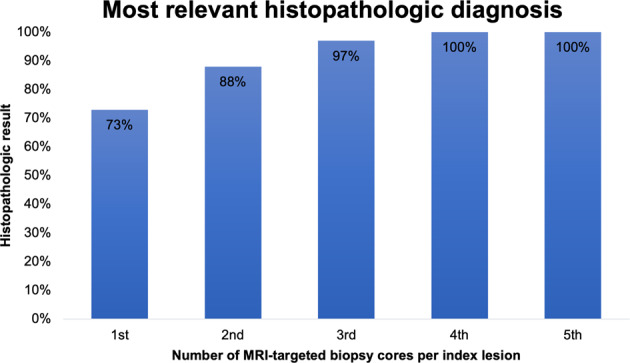
Distribution of the first MRI-targeted biopsy core revealing the most relevant histopathologic diagnosis of the index lesion. In the study population, the most relevant histopathologic result was detected with one biopsy core in 73%, with two biopsy cores in 88%, with three biopsy cores in 97%, and with four biopsy cores in ≈100% of cases. A fifth biopsy core did not add to the detection of more relevant histopathologic findings.

In 331 cases (73%), the first of the MRI-targeted biopsy cores taken of the index lesion detected on mpMRI already revealed the most relevant histopathologic diagnosis, whereas in 66 patients (15%) the second and in 39 patients (9%) the third biopsy core showed the most relevant histopathologic diagnosis. In 13 index lesions (3%), the fourth MRI-targeted biopsy core and in only two index lesions (0.5%) the fifth MRI-targeted biopsy core detected the most relevant histopathologic diagnosis. The distribution of the first MRI-targeted biopsy core revealing the most relevant histopathologic diagnosis is shown in Fig. 2.

### Effects of different covariates on the number of the first MRI-targeted biopsy core revealing the most relevant histopathologic diagnosis of the index lesion

In lesions assigned a PI-RADS score of 5 or 4, the first biopsy core already revealed the most relevant histopathologic diagnosis in 123 cases (69%) and 113 cases (68%), respectively. In PI-RADS score 5 lesions, the second MRI-targeted biopsy core showed a more relevant histopathologic finding in 34 cases (19%) and the third MRI-targeted biopsy core showed a more relevant result in 19 cases (11%). Similarly, in PI-RADS score 4 lesions, the second MRI-targeted biopsy core showed a more relevant histopathologic finding in 25 cases (15%) and the third MRI-targeted biopsy core in 19 cases (11%). Three patients (2%) with a PI-RADS score 5 index lesion and 10 patients (6%) with a PI-RADS score 4 index lesion benefitted from a fourth or fifth MRI-targeted biopsy core revealing a more relevant histopathologic finding. Regarding PI-RADS score 3 lesions, in 51 cases (86%) the first, in 5 cases (8%) the second, in 1 case the third (2%), and in 2 cases the fourth (3%) MRI-targeted biopsy core revealed the most relevant histopathologic diagnosis. All most relevant histopathologic diagnoses were obtained by the first or second MRI-targeted biopsy core in lesions rated as PI-RADS 2.

Mean PSA density was similar in the different subgroups of the study population and ranged from 0.19 to 0.21 ng/ml in all cases regardless of the number of MRI-targeted biopsy cores needed to reveal the most relevant histopathologic diagnosis.

The smallest mean diameter of MRI-targeted prostate index lesions was found in the group in which the fifth biopsy yielded the most relevant histopathologic diagnosis (11.8 ± 0.3 mm). Mean lesion size was 15.6 ± 6.6 mm in cases in which the first, 17.0 ± 8.2 mm in cases in which the second, 16.1 ± 6.2 mm in cases in which the third, and 13.9 ± 8.2 mm in cases in which the fourth MRI-targeted biopsy core was the one that yielded the most relevant histopathologic diagnosis.

In the PZ, the most relevant histopathologic diagnosis from MRI-targeted biopsy cores was found in the first biopsy core in 224 cases (72%), in the second in 50 cases (16%), in the third in 26 cases (8%), in the fourth in 9 cases (3%), and in the fifth in 1 case (<0.5%). In the TZ, the most relevant histopathologic diagnosis from MRI-targeted prostate biopsy cores was found in the first biopsy core in 107 cases (74%), in the second in 16 cases (11%), in the third in 13 cases (9%), in the fourth in 4 cases (3%), and in the fifth in 1 case (1%).

MRI-targeted biopsy cores obtained in the apex of the prostate revealed the most relevant histopathologic diagnosis with the first MRI-targeted biopsy core in 127 cases (72%), with the second in 29 cases (16%), with the third in 16 cases (9%), and the fourth in 5 cases (3%). Samples obtained in the midgland of the prostate revealed the most relevant histopathologic diagnosis with the first MRI-targeted biopsy core in 126 cases (72%), with the second in 26 cases (15%), with the third in 17 cases (10%), the fourth in 6 cases (3%), and the fifth in 1 case (1%). MRI-targeted biopsy cores obtained in the base of the prostate revealed the most relevant histopathologic diagnosis with the first MRI-targeted biopsy core in 78 cases (80%), with the second in 11 cases (11%), with the third in 6 cases (6%), the fourth in 2 cases (2%), and the fifth in 1 case (1%).

Table 2 shows the distribution of PI-RADS scores, PSA densities, lesion sizes, zones, and locations grouped by the first MRI-targeted biopsy core revealing the most relevant histopathologic diagnosis.

**Table 2:** Distribution of PI-RADS v2.1 scores, PSA densities, lesion sizes, zones, and locations within the prostate grouped by first MRI-targeted biopsy core revealing the most relevant histopathologic diagnosis.

	1st biopsy core (*n* = 331)	2nd biopsy core (*n* = 66)	3rd biopsy core (*n* = 39)	4th biopsy core (*n* = 13)	5th biopsy core (*n* = 2)	Overall (*n* = 451)
PI-RADS (PZ/TZ)						
score 2	28/16	2/0	0/0	0/0	0/0	30/16
score 3	32/19	5/0	0/1	0/2	0/0	37/22
score 4	97/16	22/3	16/3	8/0	1/1	144/23
score 5	66/57	21/13	10/9	1/2	0/0	98/81
PSA density (ng/ml^2^)	0.20 ± 0.06	0.20 ± 0.04	0.21 ± 0.04	0.19 ± 0.06	0.20 ± 0.00	0.20 ± 0.05
Lesion size (mm)	15.6 ± 6.6	17.0 ± 8.2	16.1 ± 6.2	13.9 ± 8.2	11.8 ± 0.3	15.8 ± 6.8
Zone						
PZ	224	50	26	9	1	310
TZ	107	16	13	4	1	141
Location						
apex	127	29	16	5	0	177
midgland	126	26	17	6	1	176
base	78	11	6	2	1	98

*N* number, *ng* nanogram, *ml*^*2*^ square milliliter, *mm* millimeter, *PZ* peripheral zone, *TZ* transition zone.

### Prediction model for the first MRI-targeted prostate biopsy core revealing the most relevant histopathologic diagnosis

**Fig. 3:**
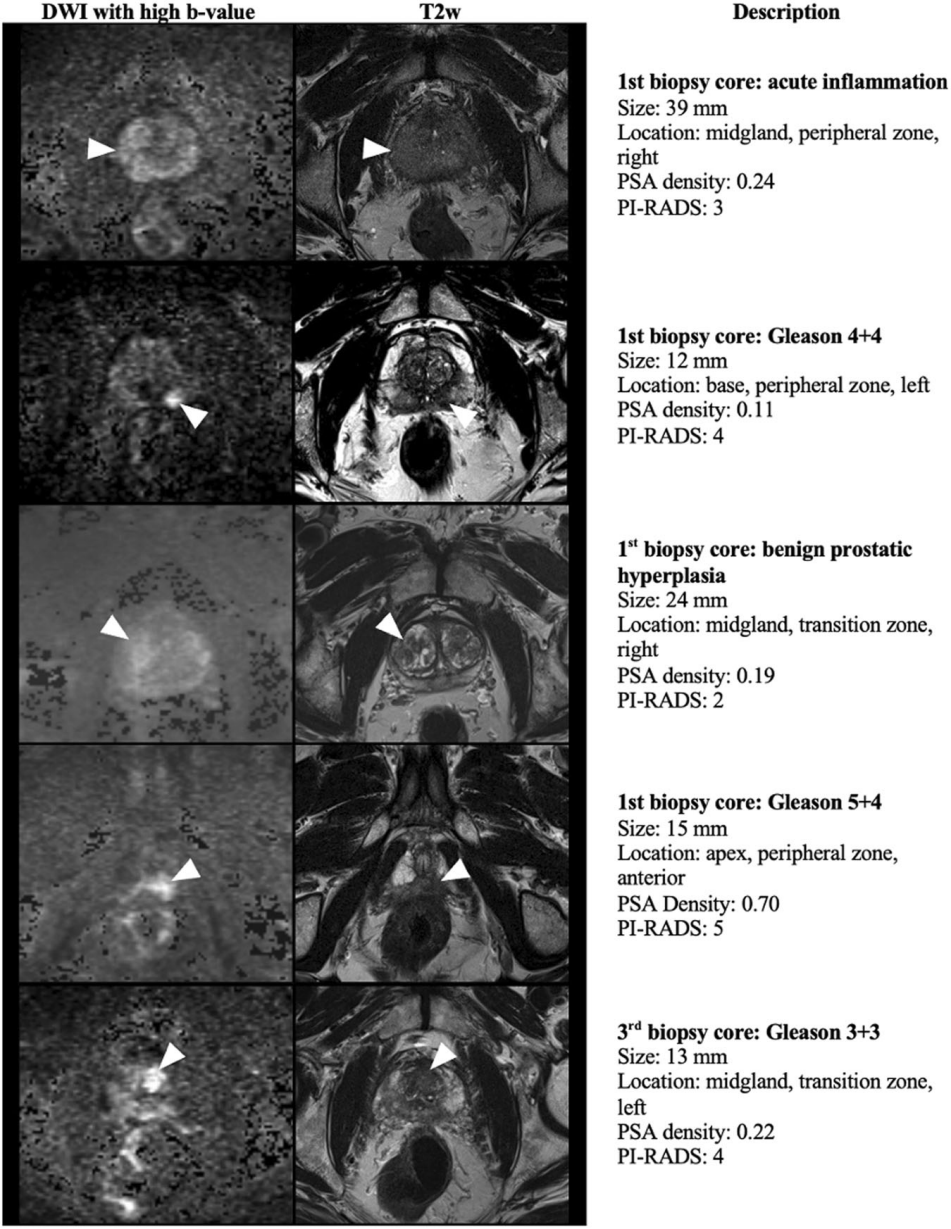
Five examples of MRI findings in patients in whom the first MRI-targeted prostate biopsy core rendered the most relevant histopathologic diagnosis despite diverse lesion characteristics in terms of PI-RADS score, PSA density, lesion size, zone, and location. 3-Tesla mpMRI images: axial DWI (left) and axial T2w (right). PCa prostate cancer.

**Fig. 4:**
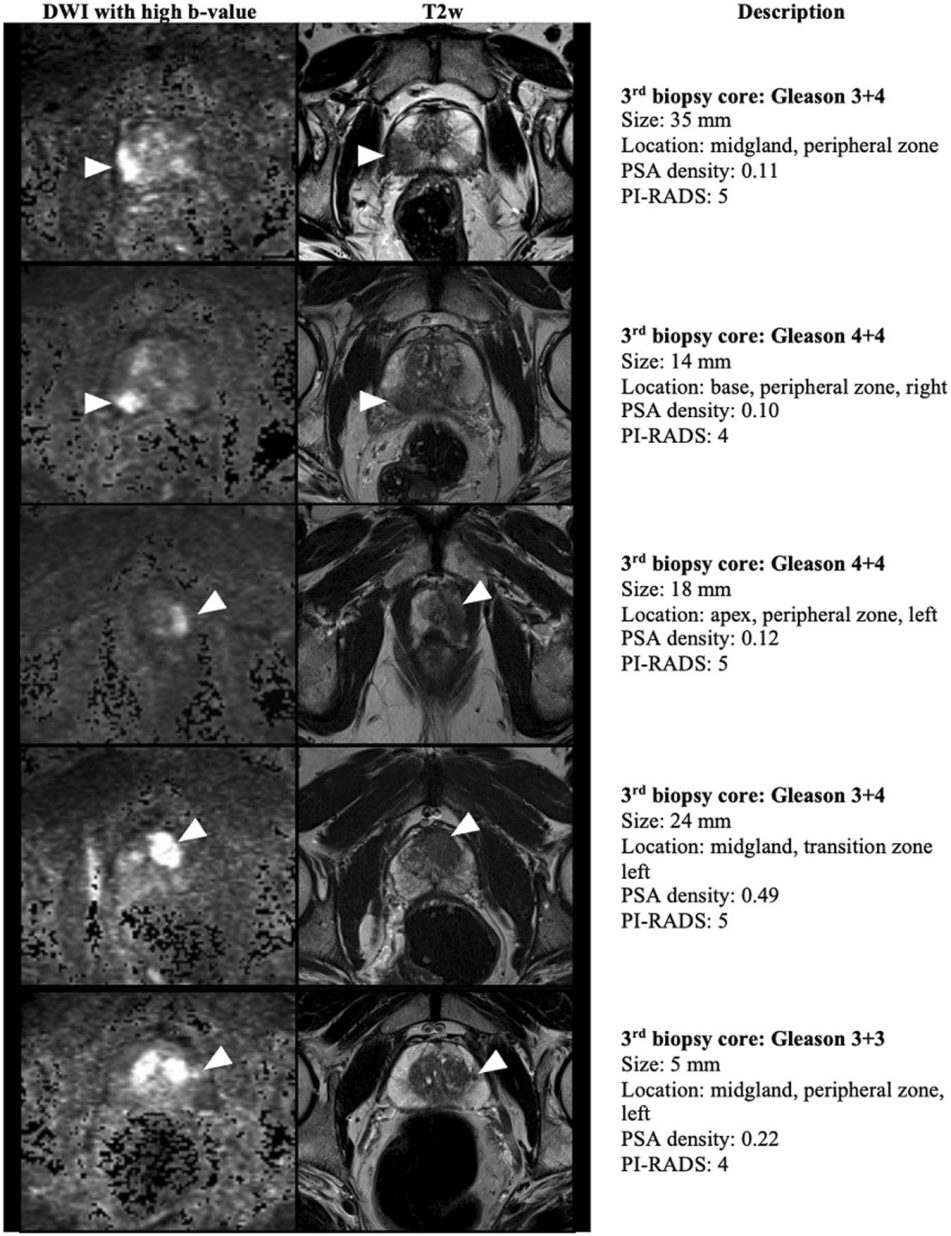
Five examples of MRI findings in patients in whom the third MRI-targeted prostate biopsy core rendered the most relevant histopathologic diagnosis despite diverse lesion characteristics in terms of PI-RADS score, PSA density, lesion size, zone, and location. 3-Tesla mpMRI images: axial DWI (left), and axial T2w (right). PCa prostate cancer.

A total of 261 patients had exactly three MRI-targeted prostate biopsy cores sampled from the index lesion detected on mpMRI. To estimate the potential to correctly predict the first MRI-targeted biopsy core revealing the most relevant histopathologic diagnosis, we used this patient subgroup to train and validate a prediction model based on the PI-RADS score, PSA density, lesion size, zone, and location as covariates. The nonparametric Bayes classifier correctly predicted the first MRI-targeted biopsy core which would deliver the most relevant histopathologic diagnosis in 79% of the subjects in the training dataset. The naïve Bayes method correctly predicted 72% of cases. Examples of MRI findings in patients in whom the first and third, respectively, MRI-targeted prostate biopsy core rendered the most relevant histopathologic diagnosis despite diverse lesion characteristics in terms of PI-RADS score, PSA density, lesion size, zone, and location are shown in Figs. 3 and 4.

## Discussion

In this study, we analyzed how many biopsy cores are needed to obtain the most relevant histopathologic diagnosis of the index lesion detected on mpMRI of the prostate and built a prediction model to forecast this number. The majority of 73% of the most relevant histopathologic diagnoses were detected by the first MRI-targeted biopsy core, 15% by the second, and 9% by the third. In less than 4% of cases, the fourth or the fifth MRI-targeted biopsy core rendered a more relevant histopathologic finding. Additionally, the detection of csPCa did not improve when a fourth or fifth MRI-targeted biopsy core of the index lesion was obtained. The Bayes classifier correctly predicted which MRI-targeted biopsy core yielded the most relevant pathologic diagnosis of the index lesion in four out of five cases.

Prostate biopsies are obtained for histopathologic verification in patients with suspected PCa, but periprocedural complications including infection, hematospermia, hematuria, and rectal bleeding may occur, and patients may need rebiopsy after initially negative biopsy [21, 44]. The risk of acute prostatitis is reduced by oral or IV antibiotics, and periprostatic anesthesia reduces pain during biopsy [45, 46]. As systematic prostate biopsy alone is associated with the risk of undersampling csPCa in about 30% of cases, it is usually combined with targeted biopsy to achieve the best cancer detection rate [47, 48]. Compared with systematic biopsy, MRI-targeted prostate biopsy performs better in detecting cancer that needs to be treated while avoiding the diagnosis of cnsPCa [49]. In our study, around 60% of MRI-targeted index lesions rated PI-RADS 4 or 5 showed cancerous tissue, which is in accordance with other studies. For example, Walker et al. found that the cancer detection rate for PI-RADS 4 and 5 lesions was 44 and 80%, respectively, and Hofbauer et al. showed that the cancer detection rate for PI-RADS 4 and 5 lesions was 49 and 77%, respectively [50, 51].

However, the optimal number of MRI-targeted biopsy cores needed to characterize lesions suspicious for prostate cancer is still unclear. For example, Ploussard et al. recommend that at least four MRI-targeted biopsy cores should be obtained in PI-RADS score 3 lesions and at least three in PI-RADS score 4 or 5 lesions [52]. Conversely, Kenigsberg et al. demonstrate that two biopsy cores can already identify about 88% of csPCa and that only little improvement in cancer detection is achieved when further biopsy cores are sampled [36]. In our study, 97% of the most relevant histopathologic diagnoses of prostate index lesions were detected by the first three MRI-targeted biopsy cores. We also found that additional MRI-targeted biopsy cores did not add to the detection rate of csPCa. Our results are in accordance with a study of Calio et al., who report that an increasing number of MRI-targeted biopsy cores is also associated with a higher rate of detection of cnsPCa, which may put patients at risk for overtreatment [38].

As most relevant histopathologic findings were detected by the first three MRI-targeted biopsy cores, we created a prediction model to forecast the first MRI-targeted biopsy core revealing the most relevant histopathologic diagnosis of the index lesion using a set of variables determined before biopsy. Our Bayes classifier correctly predicted the first relevant MRI-targeted biopsy core of the index lesion detected on mpMRI in 79% of cases. However, neither the PI-RADS score, PSA density, lesion size, zone, nor location independently influenced the prediction of the first MRI-targeted biopsy core. This result might be attributable to the high detection rate of the most relevant histopathologic diagnosis by the first MRI-targeted biopsy core. While most relevant histopathologic findings were detected using MRI-targeted biopsies, without the additional cores obtained by systematic biopsy of the same region, csPCa would have remained undetected in 2% of all patients in our study population. Thus, even though MRI-targeted tissue sampling in biopsy-naïve patients improves the detection of csPCa, it seems that it cannot avoid the need for systematic biopsy [53]. This finding is in accordance with a study of Johnson et al., who found that mpMRI may potentially miss up to 35% of csPCa and up to 20% of high-grade PCa compared to the reference standard of radical prostatectomy whole-mount specimens [54]. Thus, biopsy should still be considered in patients with persistent clinical suspicion of PCa despite negative mpMRI (PI-RADS 1 and 2).

The identification of possible predictors of the number of biopsy cores needed would be extremely helpful in clinical routine. For example, Chen et al. have shown that covariates including age, PSA, abnormal digital rectal examination, and PSA density are independent predictors of csPCa [55]. Recently, another study has confirmed that the European Randomized Study of Screening for Prostate Cancer risk calculator 3/4 (ERSPC-RC3/4) is performing well regarding the probability of csPCa [56]. Still, none of these models have been used to investigate whether covariates including PI-RADS score, PSA density, lesion size, zone, and location, when assessed preinterventionally, are predictive of the first MRI-targeted biopsy core revealing the most relevant histopathologic diagnosis. To our knowledge, the study we present here is the first systematic investigation of possible factors influencing the number of biopsy cores needed to establish the most relevant histopathologic diagnosis for both cancerous and noncancerous lesions. Also, for the first time, our systematic analysis and prediction model of influencing factors use the updated PI-RADS version 2.1, which is slightly different in diagnostic performance and interreader variability compared with its predecessor [57, 58]. In our patient population, the trained classifier correctly predicted the first MRI-targeted biopsy core revealing the most relevant histopathologic diagnosis in 79% of cases. Even though this result may be encouraging, prediction models for MRI-targeted biopsy may be limited by the fact that most relevant histopathologic findings of prostate index lesions are already detected by the first MRI-targeted biopsy core. To avoid underdiagnosing csPCa, we believe that three MRI-targeted biopsy cores should be obtained in all cases regardless of what is predicted by the model.

## Conclusion

Almost all most relevant histopathologic diagnoses of prostate index lesions detected on mpMRI can be obtained by sampling three MRI-targeted prostate biopsy cores, whereas more than three MRI-targeted prostate biopsy cores did not provide clinically relevant additional information in our study. Determination of covariates including the PI-RADS score, PSA density, lesion size, zone, and location before biopsy can help in predicting the first MRI-targeted biopsy core that will reveal the most relevant histopathologic diagnosis. However, the trained classifier only predicts four out of five cases correctly. Therefore, at least three MRI-targeted biopsy cores should be obtained regardless of the results for these covariates and the forecast of the prediction model.

## Limitations

Our study is limited by the use of a retrospective dataset. The number of obtained MRI-targeted biopsy cores could have been biased by decisions made by the interventional urologist during the procedure, e.g., if an index lesion represented cancerous tissue, which might be easier to obtain than inflamed tissue, or if the length of the core was not sufficient. Most relevant histopathologic findings are likely to be detected when at least three MRI-targeted biopsy cores are obtained; however, in theory, the “true” reference histopathologic diagnosis can only be obtained by prostatectomy or long-term clinical patient follow-up. Moreover, as most relevant histopathologic findings are already detected by the first MRI-targeted biopsy core, prediction models may have limited informative value. The predictive value of PI-RADS could have been cached as mostly lesions rated ≥ PI-RADS 3 were targeted. Finally, the clinician’s decision to perform MRI-targeted biopsies of lesions rated PI-RADS 2 by the uroradiologist might have been biased by his own rating of the prostate mpMRI findings (“interreader agreement”), even though shared decision making with these patients was always ensured.

## Data Availability

The datasets generated during and/or analyzed during the current study are available from the corresponding author on reasonable request.
